# 3 months transit time to Mars for human missions using SpaceX Starship

**DOI:** 10.1038/s41598-025-00565-7

**Published:** 2025-05-22

**Authors:** Jack Kingdon

**Affiliations:** Physics Department, University of California, Santa Barbara, 93106 USA

**Keywords:** Aerospace engineering, Astronomical instrumentation

## Abstract

Historically, spacecraft have followed trajectories that took between six and nine months to reach Mars, using traditional chemical propulsion on roughly Hohmann transfers. It is commonly believed that advances in propulsion technology, such as nuclear thermal or VASIMR, are necessary to reduce that transit time. In this paper, we show the feasibility of transit to Mars using the SpaceX Starship taking 90 days. We outline two trajectories that reduce each transit to between 90 and 104 days each way. These trajectories are within NASA career radiation limits, while 180-day trajectories are not.

## Introduction

A key challenge for human missions to Mars is the lengthy transit time (6–9 months^1,2^) using low-energy trajectories. Such durations not only complicate mission design and technology requirements, but also raise questions about crew survivability and overall success. Studies^3,4^ regularly emphasize how time in interplanetary space increases mission risks in multiple ways: crew health, radiation, resupply logistics, and psychological factors. Notably, bone health^5^ risks and cancer risk^4^ scale roughly linearly with time in space. As a result, shortening the Mars transit window has emerged as a priority, with numerous studies proposing advanced propulsion options-such as VASIMR engines operating with high specific impulse^6^, nuclear thermal approaches to achieve faster transfers^7^ and laser propulsion^8^. Concurrently, NASA’s Journey to Mars and other policy frameworks have emphasized the need for safer, more rapid human missions^9^. Certain NASA presentations^10^ even claim nuclear powered propulsion systems are the only primary contenders for human Mars missions. This study challenges the prevailing assumption that advanced propulsion technologies, such as nuclear thermal or VASIMR, must be employed for rapid human transit to Mars. By demonstrating that 90-day transfers are feasible using existing chemical propulsion technology, our work provides a near-term, practical alternative to proposed nuclear thermal or nuclear electric propulsion solutions that have low technology readiness levels and require significant regulatory approval processes^11^.

## Mission outline

**Fig. 1:**
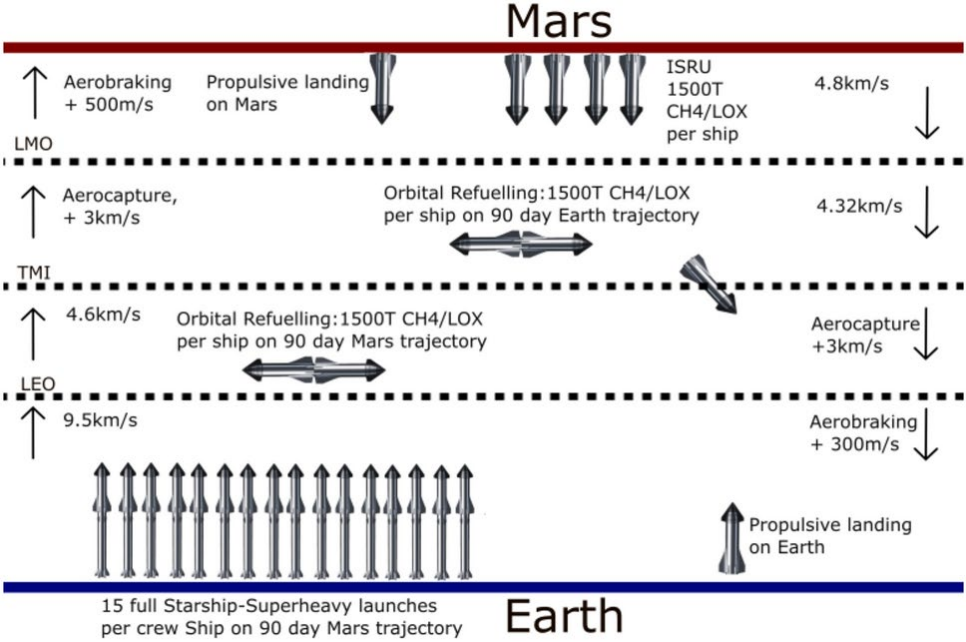
Starship 2033 mission with 90-day transit architecture. The DV cost of each step is labeled next to the arrows.

As outlined in the SpaceX Starship Mars plan^12^ the crew mission would involve four cargo Starships and two crew Starships. The crew Starships would require 15 refuels in low Earth orbit (LEO) assuming Block 2 Starship is capable of 100t (metric tonnes) to LEO^13^ and has 1500t propellant capacity. The cargo ships would be sent on longer low-energy trajectories, each requiring four refuels in LEO. The outlined mission involves $\sim$ 45 launches of Starship Superheavy (Fig. 1) which given a speculative cadence of 1000^14^ launches per year would be achievable in 2–3 weeks. If SpaceX is unable to improve launch rate beyond their current Falcon 9 cadence (15 launches/month as demonstrated in November 2024) these launches would take 2–3 months.

Upon arrival at Mars, 1500t of propellant per ship would be produced from local carbon dioxide and water ice using electrolyzers and a Sabatier ISRU (in situ-resource-utilization) reactor^12^^15^. Astronauts would explore the local area and perform science experiments. When near the return window, one crew and 3–4 cargo ships would be refuelled. All ships would launch into LMO (low Mars orbit) where the cargo ships would transfer the majority of their propellant to the crew ship until it is fully refueled. The cargo ships would then return to the surface of Mars, while the crew ship would head for Earth. This could be repeated for the other crew ship.

## Trajectory

### Assumptions and methods

We took the dry mass of an empty tanker Starship to be 100t^16^ and the mass of a crew habitat to be 63t (see Appendix A). We took the tanks to have 1500t of propellant capacity, of which 1170t is LOX (liquid Oxygen) and 330t is LCH4 (liquid Methane)^13^. SpaceX aims to reach a specific impulse of 380 s^17^ with the Raptor Vacuum engine, but this is a highly ambitious goal that may not be achieved. There is no recent public information on specific impulse other than a claim that the ISP will reach 380 s in a few years, however Elon Musk has claimed that the 2021 version of Raptor Vacuum had an ISP of 378 s^18^. We can estimate performance based on known parameters such as chamber pressure^19^ and expansion ratio^20^ using NASA CEA^21^. We end up with specific impulses between 367 s and 384 s, roughly backing up public info—we do not know temperatures of propellants upon combustion chamber entrance, exact chamber pressure during vacuum operation, quantity of film cooling and throat diameter. We also note that the expansion ratio has likely increased since 2020^17^. We take 370 s as a conservative estimate based off public information^18^ and 380 s as the future case. All burns are assumed to be done with only the Raptor vacuum engines as the extra thrust from the sea level engines is not necessary during orbital maneuvers.

The trajectories were calculated with patched conics 2-body approximations using the poliastro python library^22^^23^. As is standard in preliminary Mars mission architecture studies^1^, we employ Lambert-arc solutions for Earth-Mars transfers, effectively treating the spacecraft’s heliocentric leg as a two-body problem (Sun + vehicle), and each hyperbolic planet exit/entry as a two-body problem (Earth/Mars + vehicle). Higher-order multi-body perturbations are negligible for initial DV (delta-velocity) estimates and remain consistent with the approach used historically by NASA. We iterated the poliastro Lambert-arc solver over 1-day timesteps in the 2030 s to look for the lowest ejection DV solutions in the Sun reference frame.

We then calculated the C3 (characteristic energy) of said trajectory when propagated to edge of Mars/Earths sphere of influence. Then a hyperbolic trajectory with that C3 could be calculated, and thus the ejection/arrival DVs could be calculated. The Delta-V for 5 day timesteps in both departure date and time of flight in the 2030 s is plotted in two Porkchop plots below (Figs. 4, 5). Red colors indicate more energetically favorable transits. Our model predicts transits with delta-velocity and arrival characteristics that closely align with those presented by Wooster et al.^24^—e.g. an Earth ejection DV of 3.57 km/s for a 180-day 2033 trajectory matches their $\sim$ 3.6 km/s.

## Earth to Mars

We outline two 90-day Earth to Mars transit opportunities in the mid 2030s.

**Fig. 2:**
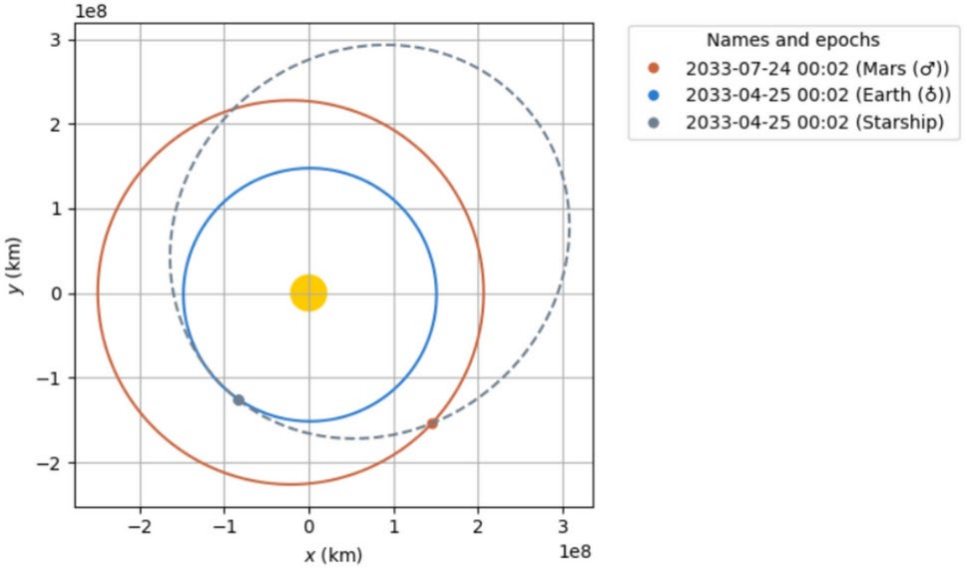
2033 90 day transit.

**Fig. 3:**
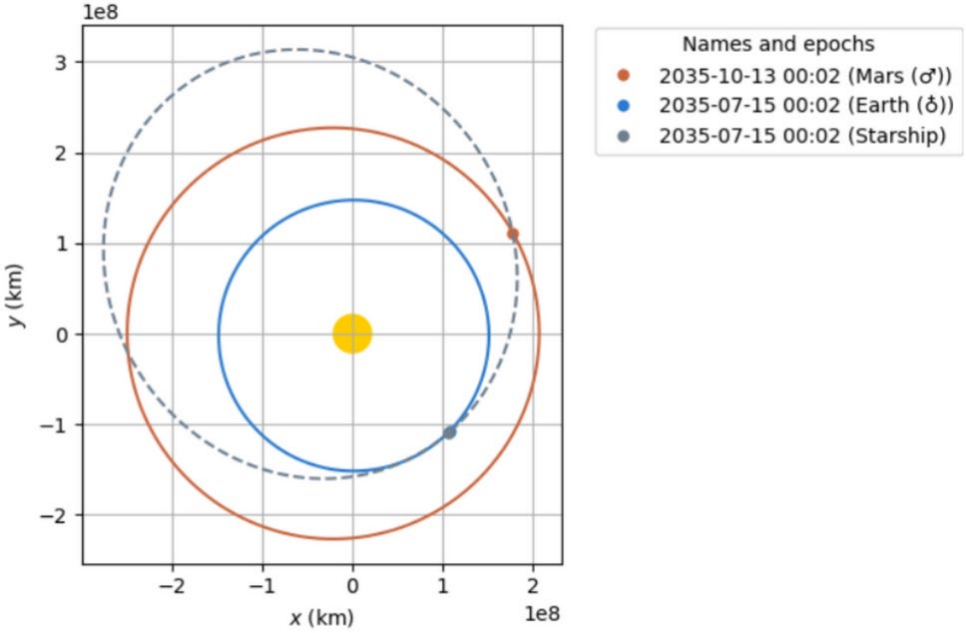
2035 90 day transit.

- 2033 trajectory: Ejection from Earth on 2033-04-30 with $\sim$ 4.6 km/s from a 150 km circular LEO. Mars aerocapture $\sim 90$ days later (Fig. 2).
- 2035 trajectory: Ejection on 2035-07-15, also $\sim$ 4.6 km/s from LEO, with aerocapture at Mars $\sim$ 90 days later (Fig. 3).

The respective characteristic energies ($C_3$) for each trajectory are roughly $31.5 \; km^2/s^2$ and $32 \; km^2/s^2$. There are no trajectories with similarly low ejection DV until 2048-04-08 with 4.60 km/s. Each trajectory leaves the Starship with $\sim$ 3.5 km/s of DV, of which 0.5 km/s^25^ (Appendix B) is used for the landing burn and the other $\sim 3 \; km/s$ is used for a deceleration burn near Mars entry to reduce aeroloads during aerocapture (Fig. 6). For the both trajectories, we assume a deceleration burn 400 s prior to periapsis, of which $\sim 200 \; m/s$ is lost to non-optimal oberth effect. For instance, on the 2035 trajectory with 380s ISP, the arrival speed without a deceleration burn would be 9.73 km/s, but is 6.87 km/s with it. This leaves 1.85 km/s to be shaved off by Mars aerocapture. In each case, a 370s ISP Starship takes the same time to reach Mars, but aerocaptures at a higher velocity due to reduced DV for the braking burn.

**Fig. 4:**
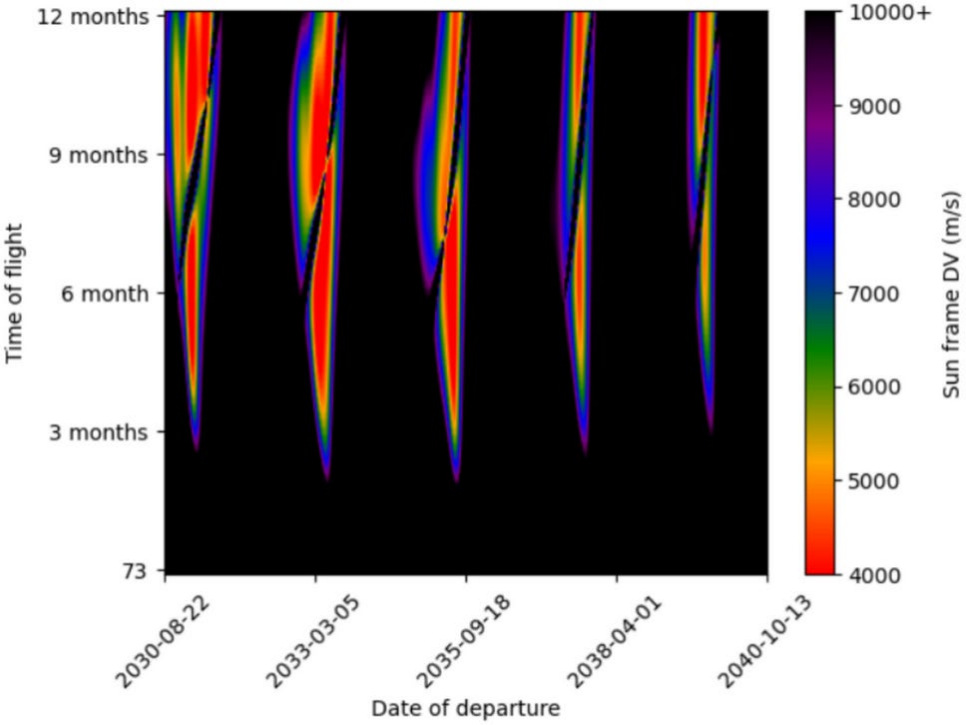
Earth to Mars Porkchop plot for the 2030s.

**Fig. 5:**
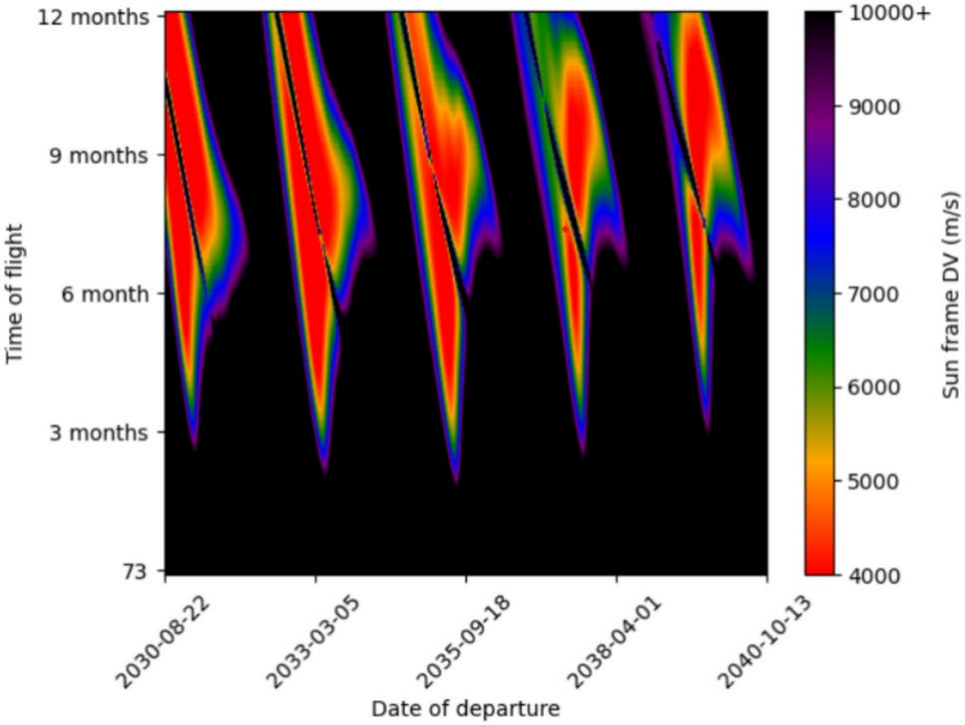
Mars to Earth Porkchop plot for the 2030s.

**Fig. 6:**
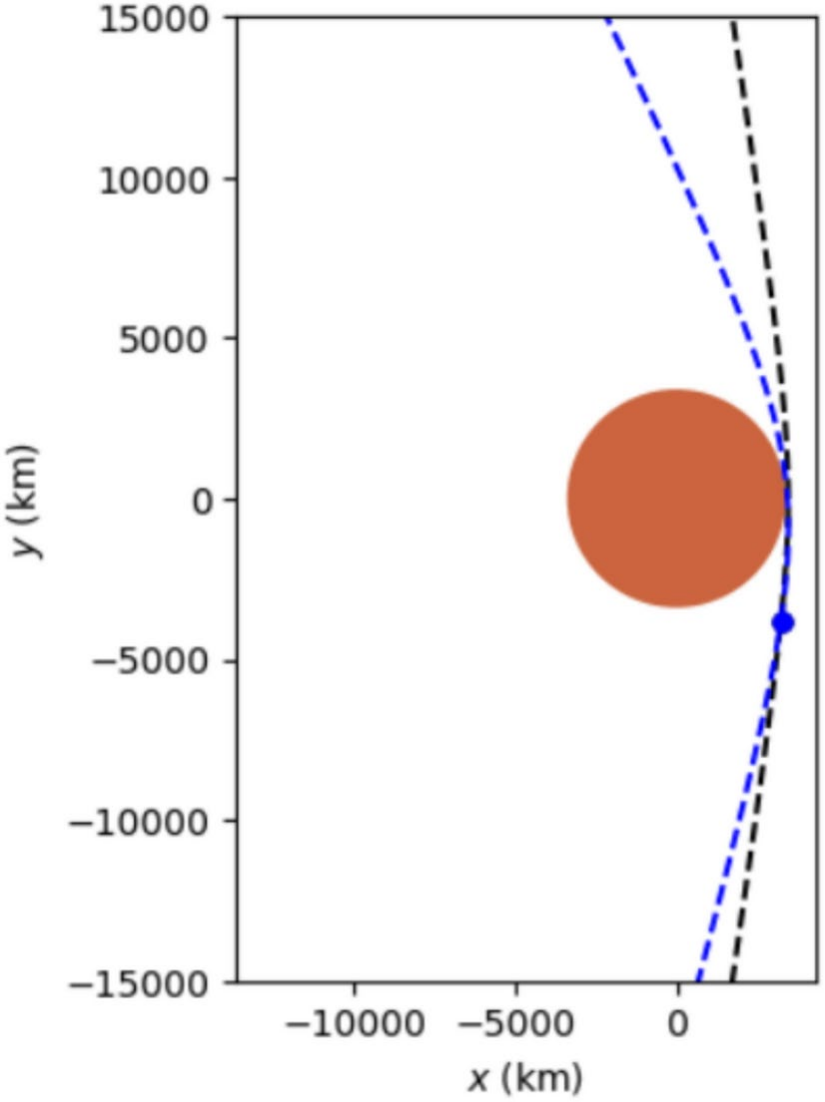
Arrival at Mars on 2035-10-13. The blue dotted hyperbola is trajectory after deceleration burn, and the blue dot is where the deceleration burn takes place.

### Mars to Earth

**Fig. 7:**
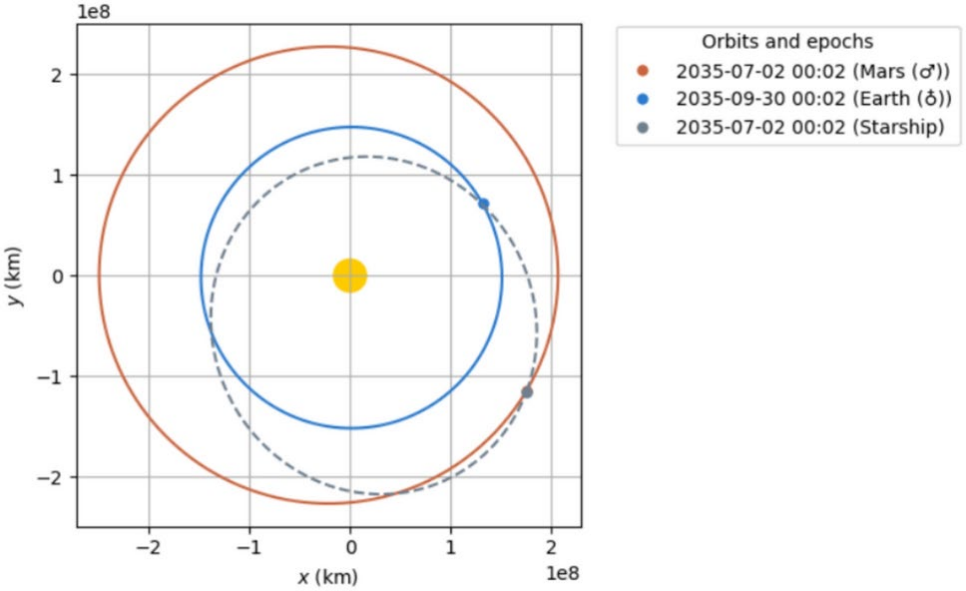
2035 90 day return.

**Fig. 8:**
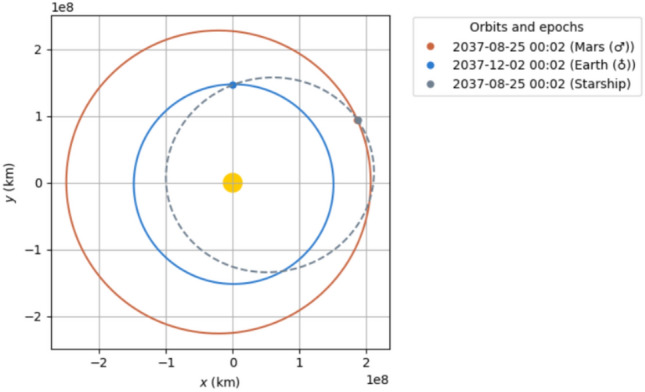
2037 104 day return.

Upon launch from Mars, the crew Starship enters LMO and is refueled by the empty cargo Starships also fueled by ISRU on the surface of Mars. Each tanker flight may deliver approximately $300 \, \text {t}$ of propellant if the Mars ascent $DV$ is about 4800 m/s^26^. Approximately 4–5 flights will be needed for full refueling. As with the Earth to Mars trajectories, some propellant is used for a deceleration burn prior to Earth arrival. We identify only one suitable 90-day return in 2035—on 2035-07-02 (Fig. 7), requiring a $DV$ of about $4.32 \, \text {km/s}$ for ejection from LMO. Summer/Autumn 2037 offers a 90-day route (2037-08-31, requiring $\sim 5.87$ km/s) but yields a dangerously high Earth arrival velocity ($\sim 14.1 \, \text {km/s}$ at periapsis). Instead, we recommend a 104-day trip starting 2037-08-23 (Fig. 8), arriving at Earth on 2037-12-05 at a periapsis velocity of about $12 \, \text {km/s}$—closer to typical lunar^27^ or Hohmann-like reentry energies and presumably within Starship’s aerodynamic/thermal design limits^28^.

### Boiloff

The cryogenic LOX & LCH4 propellant stored in the main tanks of the Starship used in the deceleration burns must survive the 3 month transit. Starship Human landing system (HLS) is contracted to land the next humans on the Moon (by NASA) and requires 100-day loiter time in cislunar near rectilinear halo orbit. The vehicle, with modifications, can store cryogenic propellant for extended periods of time in deep space. The HLS modifications preclude atmospheric entry, thus we calculate the expected thermal properties without these modifications on Block 2 Starship. The thermally optimal attitude is where the Starship points directly at the Sun to both reduce the area absorbing solar radiation, and to insulate the main tanks with the crew cabin. Any propellant in the header tanks could be moved to the main tanks during cruise and moved back for landing^29^.

**Fig. 9:**
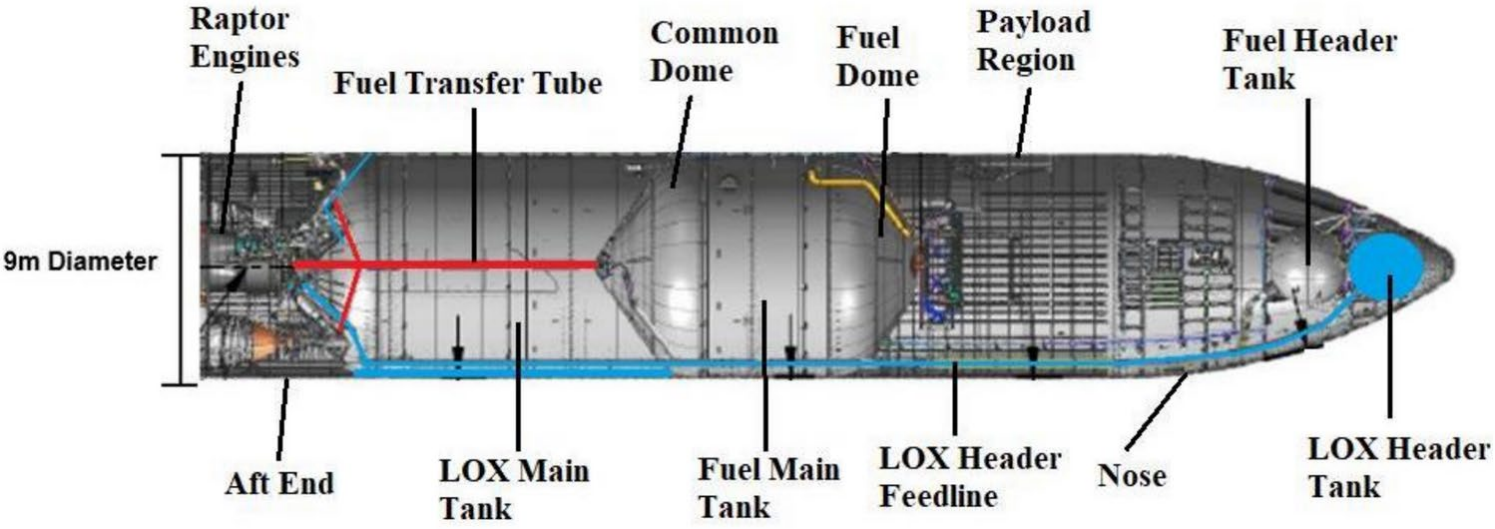
Starship internal diagram^30^. The astronaut habitat will be placed in the payload region.

Both tanks will nominally be pressurized to 6 bar^31^ where methane has a boiling point of 140 K^32^ and oxygen at 113 K^33^. The methane tank externally is composed of three 1.83 m tall, 9 m diameter steel rings^34^ (Fig. 9). The oxygen tank is composed of six of these rings thus has twice the area. Half of each tank is bare 304X stainless steel, and the other half is that steel covered with proprietary ceramic thermal protection tiles. As these are proprietary SpaceX materials, we do not know their exact specifications, but they are similar to/derivatives of 304 L stainless steel and the NASA TUFI TPS^35^ material respectively and thus have very similar emissivities. The emissivity of both vary with flight wear and other parameters, but we take them to be 0.4^36^ and 0.9^37^ respectively. The average emissivity $\bar{\epsilon }$ of the Starship tank section is thus 0.65. We take the area of the methane tank to be $A_m$, the area of the oxygen tank to be $A_o$, the respective temperatures to be $T_a$ and $T_o$ and the Stefan–Boltzmann constant to be $\sigma$. By the Stefan–Boltzmann law the radiated power from the tanks each at saturation temperature is:

$$P_r = \bar{\epsilon } \sigma (A_mT_m^4+A_oT_o^4) = 0.65\sigma (310*(140^4)+620*(113^4))m^2K^4 = 8114W$$

If the methane tank is in thermal equilibrium with the oxygen tank, then the radiated power is:

$$P_r = \bar{\epsilon } \sigma T_o^4 (A_m+A_o) = 0.65\sigma *113^4*(310+620)m^2K^4 = 5596W$$

The crew cabin is assumed to be at a human friendly 290K temperature, and assuming the bottom plate facing the propellant tanks is a polished aluminium sheet of emissivity of 0.06, the emitted power is:

$$P = 0.06*\sigma *290^4*(\pi * 4.5^2)m^2K^4 = 1530W$$

into the methane tank. Conduction through the steel skin is negligible: the conductivity of stainless steel is $\sim 15 \text {W}\text {K}^{-1}\text {m}^{-1}$^38^ at 300 K, and decreases with temperature. There is a $\sim 200 \text {K}$ temperature drop from the crew cabin outer steel to the tank outer steel, which we can assume takes place over the distance of one ring segment. The steel is 9m diameter and 4mm thick^39^, thus the conducted power is $\sim 400\;W$, which is small compared to the radiated heat. As the Sun is only hitting the nose cone, solar radiation management is only a problem for the crew cabin. Thus the tanks will radiate more power out ($5 \text {kW}-10 \text {kW}$) than they receive ($\sim 2 \text {kW}$) and the cryogenic propellant will not boil off in the 90-day transit to Mars. These calculations obviously ignore radiation from sources other than the Sun. Radiation from interstellar sources is negligible, but Earth/Mars-shine along with planetary black-body emissions is nominally not negligible and contributes significantly to the engineering challenges of long-duration LEO cryogenic storage. We ignore it as the Starship spends very little time near Earth or Mars (except when the tanks are intended to be empty, or being actively filled). We acknowledge that these calculations are simplified, modeling the Starship as a cylinder, ignoring both absorption and emission from the flaps along with the challenges of crew cabin thermal management, but we believe they show Starship interplanetary transit cryogenic storage is a manageable problem.

## Reentry simulations

Both trajectories involve significant aerocapture maneuvers where the Starship enters the upper atmosphere of Earth or Mars and uses atmospheric drag to decelerate from a hyperbolic trajectory that moves faster than the respective planet’s escape velocity to an elliptical captured orbit. Because hyperbolic trajectories around planets have a lower curvature than captured orbits, the amount of time spent in the atmosphere is not long, so the necessary acceleration to capture is high.

### Assumptions and methods

We performed an approximate simulation of the Starship atmospheric entry for each entry trajectory. The hyperbolic trajectory, generated from the Lambert’s problem solution, was numerically propagated through Earth and Mars’ upper atmosphere. Earth’s atmosphere was modeled by COESA76^40^ while Mars’ used an exponential model empirically fit to NASA measurements^41^. We assumed a constant lift to drag ratio of 0.5^42^, and an inverted $60^\circ$ AoA (Angle of Attack) entry angle during the majority of the trajectory allowing for negative lift to bend the trajectory downwards. The stagnation point heat flux was calculated with the Sutton-Graves equation^43^. While far more complex reentry analysis tools exist, we found that Sutton-Graves accurately approximated the peak stagnation point heat flux for historical missions using our model. Starship was assumed to have an equivalent body radius of 4.5 m. We calibrated our model by estimating the heat flux during the Zond 5 ballistic entry and Soyuz LEO entries. Our model calculated the Zond peak convective heatflux to be $3.05 \; \text {MW}/\text {m}^2$ and the Soyuz LEO reentry peak convective flux to be $700 \; \text {kW}/\text {m}^2$ which lines up with historical data^44^. We did not calibrate the model on Starship reentries (IFT 4, 5 & 6) as public heat flux information does not exist, but our model estimates the peak convective flux during a Starship LEO reentry to be $\sim 350 \; \text {kW}/\text {m}^2$. We also estimated radiative flux during both Zond and Starship aerocapture, using empirical formulas^45^ (correctly estimating the Zond radiative flux) and found the peak radiative flux to be negligible. We do acknowledge that the complexity of radiative heating is substantial at the velocities considered for Mars and Earth aerocapture. Accurately modeling this requires resolving non-equilibrium radiation transport in a superheated shock layer composed of dissociated and ionized gas, where radiative transfer is strongly wavelength-dependent and tightly coupled to gas chemistry. Furthermore, effects such as shock layer optical thickness and reabsorption must be taken into account. Although our analysis suggests that convective heating dominates and radiative flux may be subdominant, only a full radiative-hydrodynamic simulation can confirm whether the Starship airframe and TPS can withstand the proposed aerocapture trajectories with adequate thermal margins. We acknowledge that there are large differences between capsule entries and lifting-body aerocapture entries, so a model originally validated for capsules may not yield perfectly accurate predictions for a large vehicle like Starship. However, the total hyperbolic energy that must be dissipated in our 90-day trajectory profiles is on the same order-or even somewhat lower-than that of typical LEO return missions, which are well-characterized in both historical and modern analyses. Consequently, the peak heat flux and deceleration loads Starship would encounter at these velocities are likely to remain within the envelope that simpler capsule-derived models (Sutton-Graves for heat flux) can reasonably estimate at a first-pass level.

### Conclusions

**Fig. 10:**
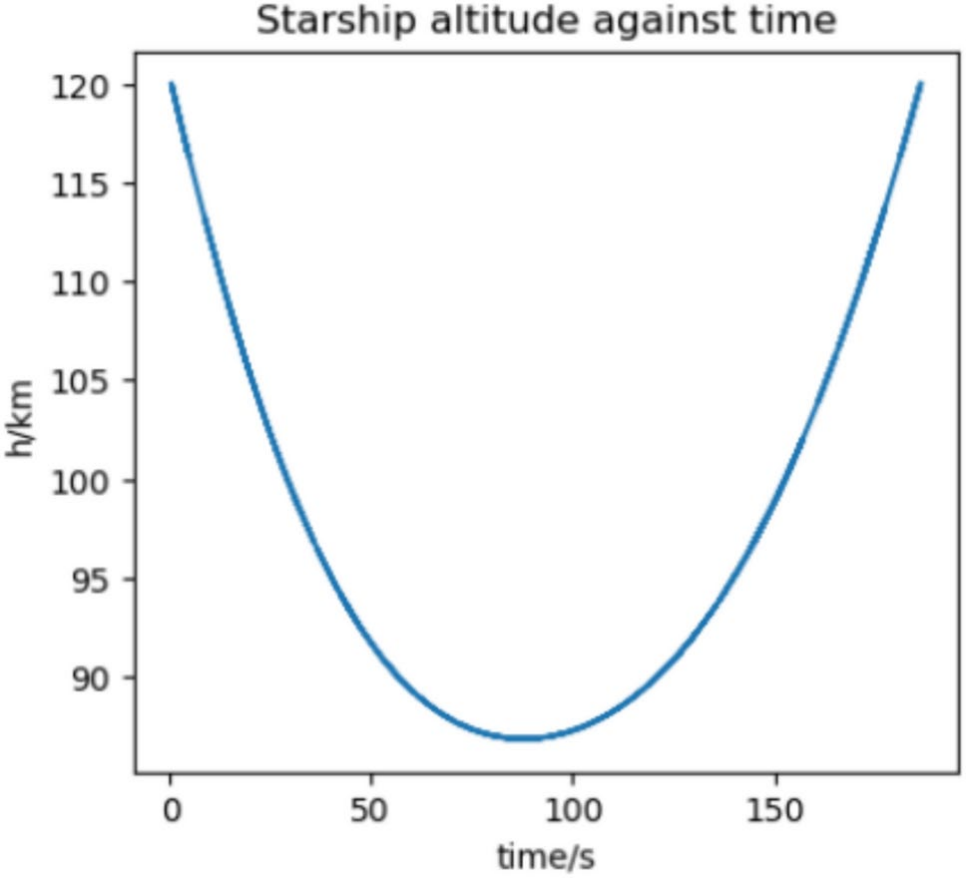
Altitude against time for the 2037 Earth aerocapture.

**Fig. 11:**
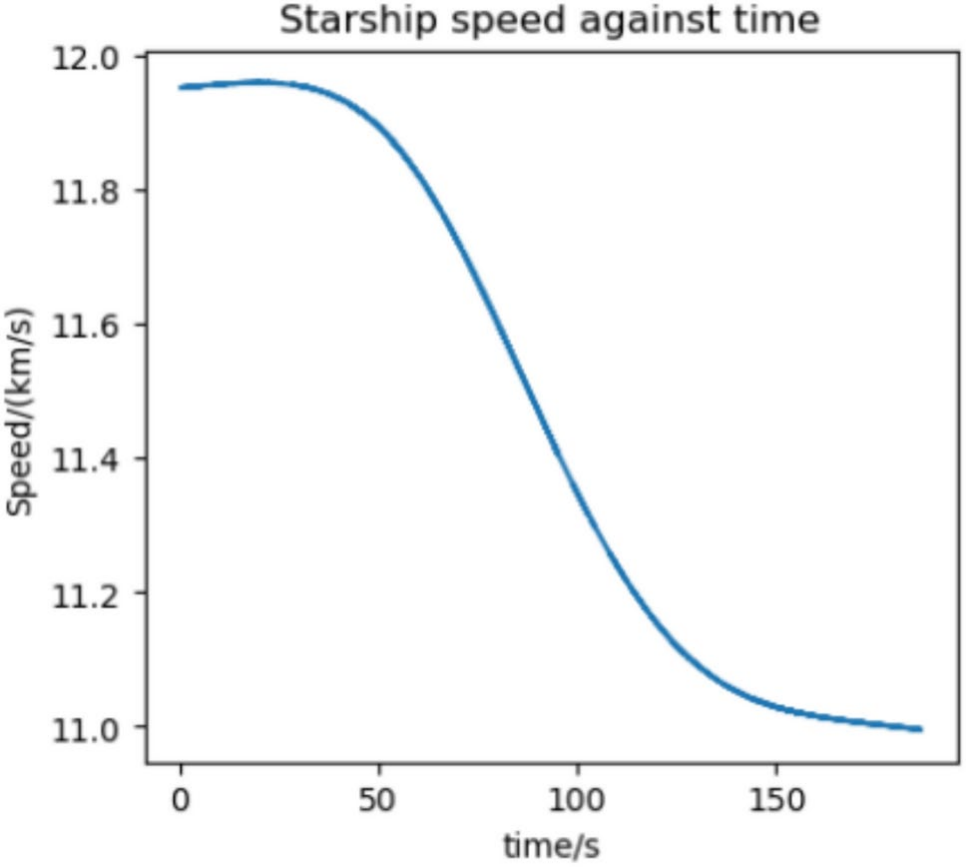
Speed against time for the 2037 Earth aerocapture.

**Fig. 12:**
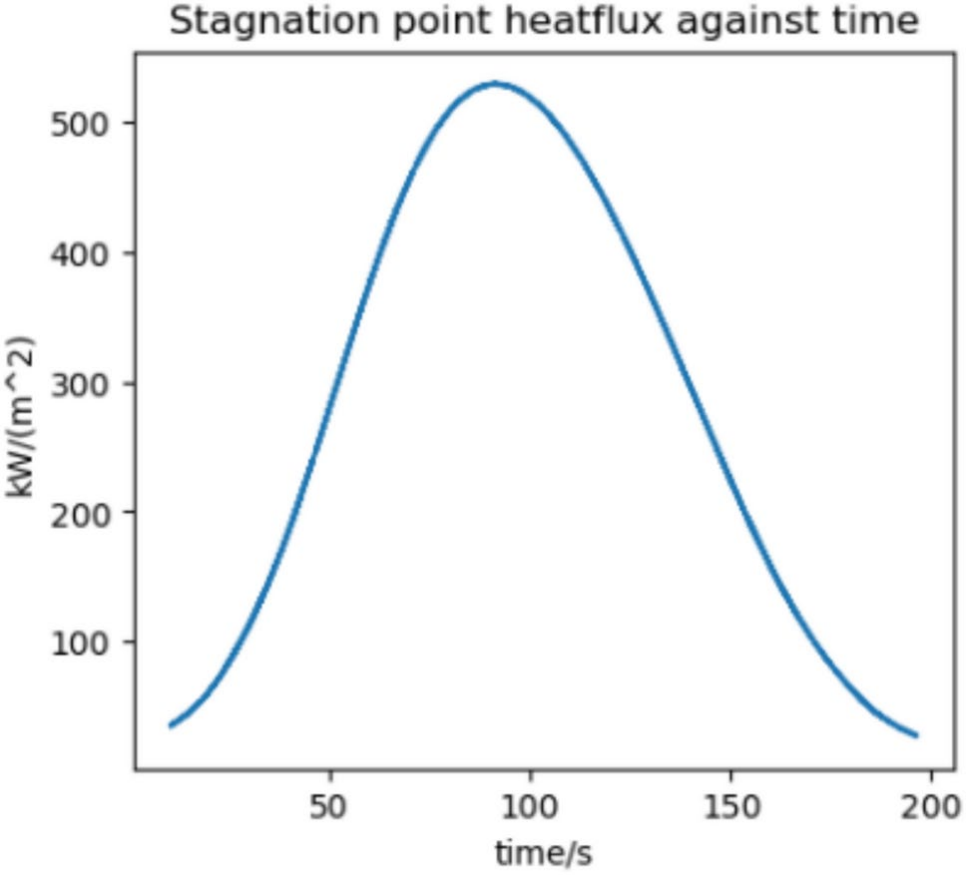
2037 aerocapture stagnation point heatflux.

**Fig. 13:**
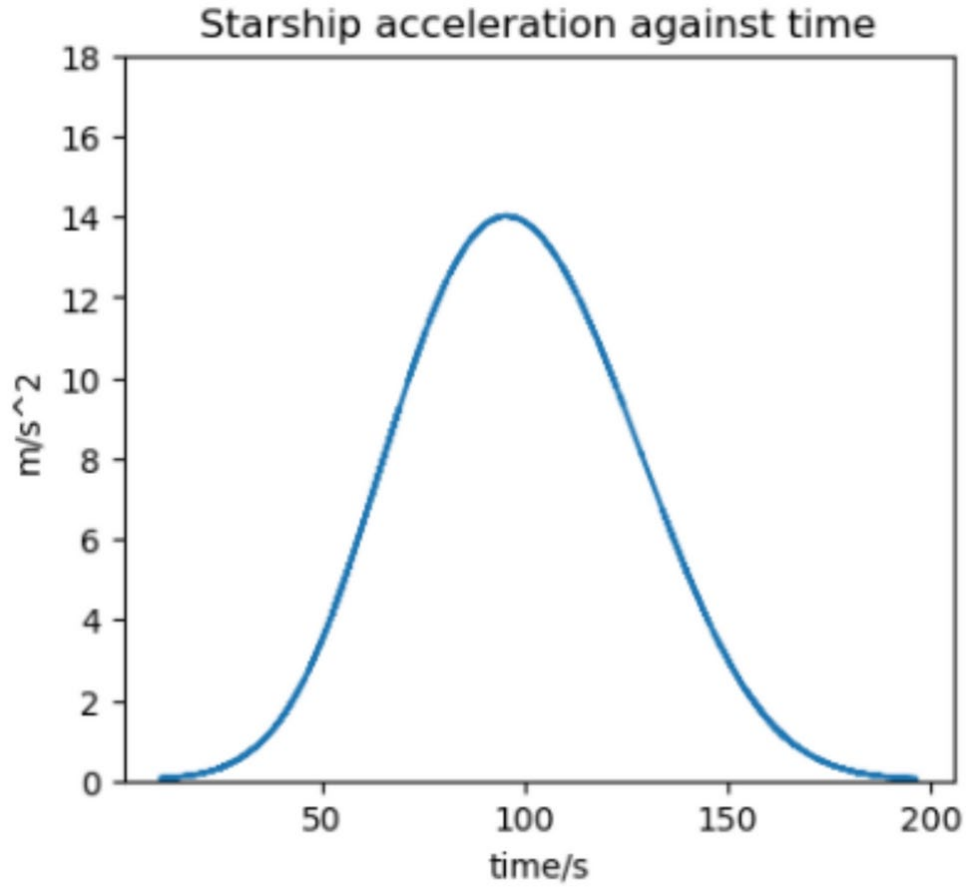
2037 aerocapture acceleration.

We estimate the trajectory with the highest flux and acceleration to be an arrival at Earth in 2037 if we assume a 370 s RVac ISP. The reentry angle is $4.2^\circ$ with a peak convective stagnation point heat flux of $530 \; \text {kW}/\text {m}^2$ (Fig. 12) and peak acceleration of 1.43G (Fig. 13). The specific energy lost is $20 \; \text {km}^2/\text {s}^2$ and the post capture orbit is 136,000 km by 90 km. The altitude and velocity plots are given by (Figs. 10, 11).

**Fig. 14:**
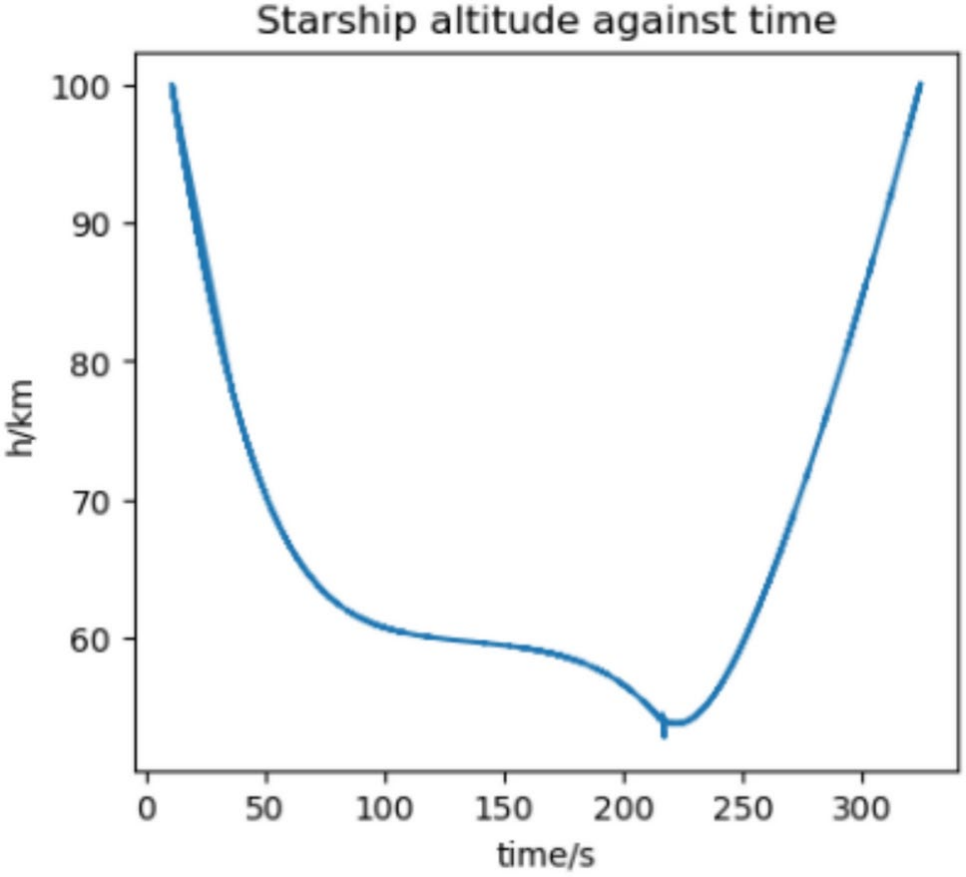
Altitude against time for the 2033 Martian aerocapture.

**Fig. 15:**
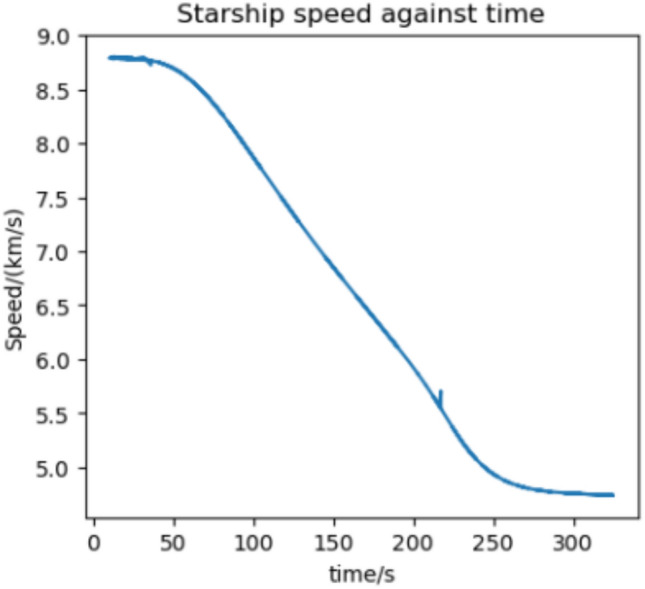
Speed against time for the 2033 Martian aerocapture.

**Fig. 16:**
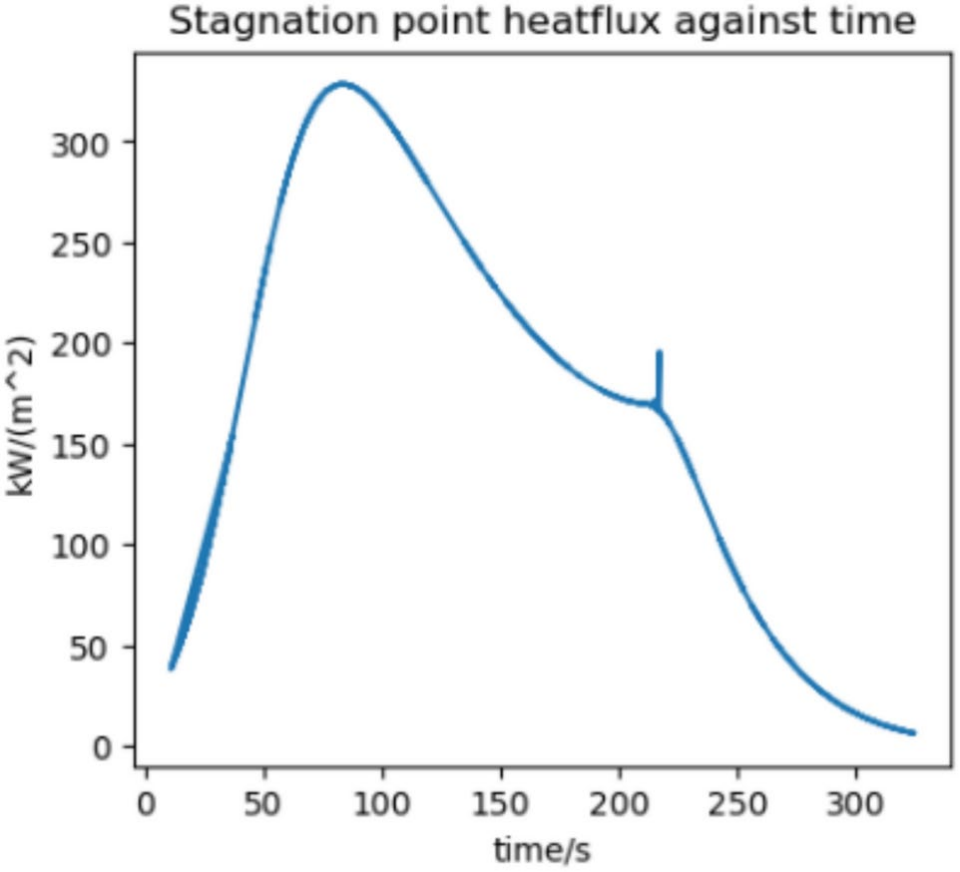
Flux against time for the 2033 Martian aerocapture.

**Fig. 17:**
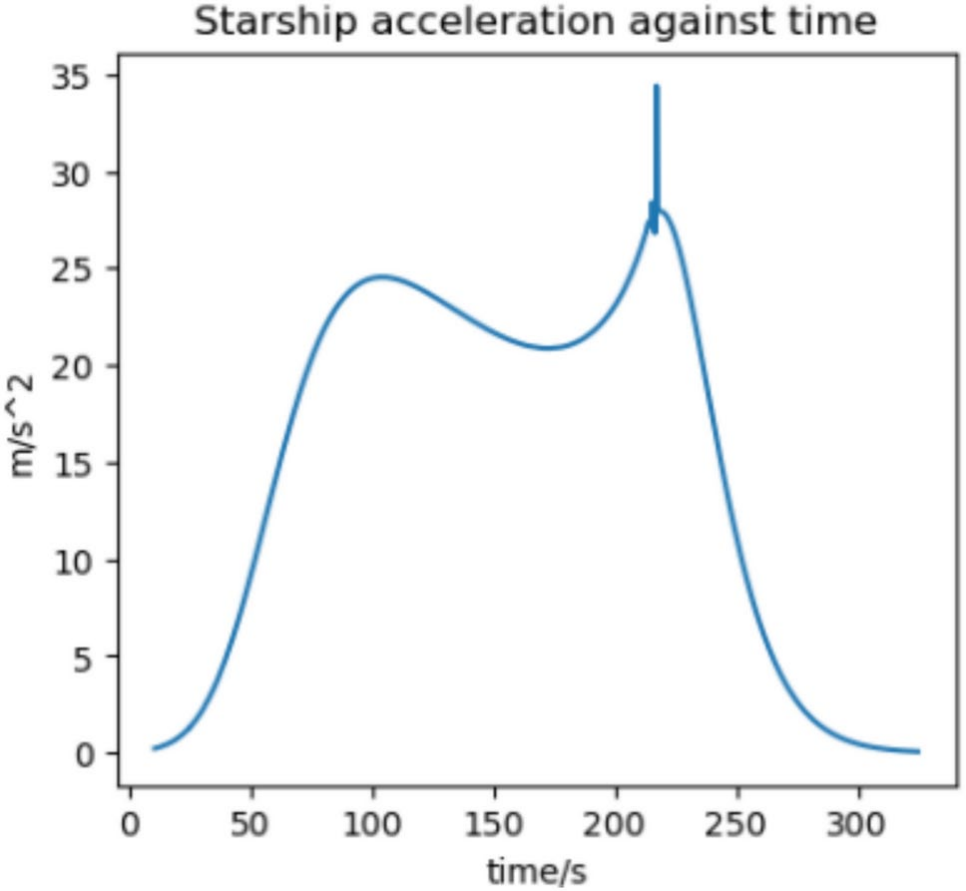
Acceleration against time for the 2033 Martian aerocapture.

The highest flux and acceleration Mars arrival is the 2033 arrival, again assuming a 370s RVac ISP. The entry angle is $7^\circ$, peak heat stagnation point convective flux is $330 \; \text {kW}/\text {m}^2$ (Fig. 16) and the peak acceleration is 3.51 g (Fig. 17). Figure 17 shows a sharp spike in acceleration around 230 s, corresponding to a flip in vehicle orientation from inverted to upright to control post aerocapture orbit parameters. This maneuver is not required, but helps relax targeting constraints. The specific energy lost is $27 \; \text {km}^2/\text {s}^2$, and the post capture orbit is 35,600 km by 25 km. The altitude and velocity plots are given by (Figs. 14, 15). We do not know the exact maximum temperature of the Starship tiles, but they are known to be coated with material similar to the Shuttle TUFI coating with a max temperature of $\sim 1920$ K and an emissivity of $\sim 0.9$^37^. At this temperature, the tiles can radiate $700 \; \text {kW}/\text {m}^2$, which is greater than the peak heat flux encountered during even the most extreme entry profiles studied here. This suggests that, in theory, the tiles possess sufficient radiative capacity to reject the incoming thermal load. However, this balance alone does not guarantee that the vehicle will survive the entry. The actual survivability of the system depends on a variety of factors beyond peak heat flux, including the duration of thermal exposure, heat soak into the underlying structure, mechanical stresses on tile retention, and the vehicle’s overall thermal and structural margins. Consequently, our model, while useful for a first-order estimate, does not capture the full complexity of the thermal environment and cannot definitively assess vehicle survivability. More detailed simulations-incorporating time-dependent conduction, radiative coupling, material response, and structural analysis-are necessary to validate the feasibility of the proposed entry maneuvers. That said, it is not unreasonable to believe that such maneuvers are within the design envelope of Starship, which is explicitly intended for high-speed hyperbolic returns from Mars^12^, with entry velocities comparable to those analyzed in this study.

## Implications of faster trajectories

### Launch cadence

Because the rapid transit trajectory demands a very high launch cadence on both Earth and Mars, requiring a dozen tanker flights in the period prior to the launch window, ground systems must operate at a pace at or beyond the current SpaceX rate (which is already extremely high). While Earth’s launch infrastructure is expected to handle rapid turnaround-thanks to growing launch demand from projects like Artemis and Starlink-the more substantial challenge lies in setting up robust ISRU facilities on Mars. While ISRU is necessary for any Mars direct style mission^46^, the faster trajectory calls for more propellant production per Earth return window and thus further burden on the ISRU system. ISRU remains an outstanding concern that SpaceX/NASA must address before human missions can take place.

### Estimated radiation dosage

The majority of radiation dosage in interplanetary space is GCR (galactic cosmic ray)^47^, with the exception of Solar storms. Modern human deep space craft employ solar storm shelters^48^, and Starship will also, thus the radiation exposure from Solar events will be mitigated. The main focus is on GCR, which cannot be easily shielded. The GCR dosage in interplanetary space has been measured at $1.84\pm 0.33$ mSv per day^47^ which does not significantly change with distance to the Sun during a Mars mission. Thus the radiation dosage for a 90-day transit is halved from a 180-day transit. Assuming significant shielding on the surface of Mars (which can be provided by local materials such as ice and Mars dirt), the radiation during transit will be the majority of the total mission radiation. This is $331\pm 54$ mSv. Taking the 104-day return trajectory in 2037 will lead to the total dose increasing to $357\pm 58$ mSv. This is below the allowed career radiation dose for astronauts^49^—1000 mSv and 600 mSv for 35-year-old men and women respectively.

### Medical impacts of faster trajectory

According to NASA’s risk models^4^, 1Sv of absorbed dose can increase the lifetime cancer risk by roughly 3–7% percent for a middle-aged adult. Thus the faster trajectory will decrease the estimated risk of GCR induced cancer from $\sim 4\%$ to $\sim 1.5\%$. A 6 month mission already puts the crew at the threshold of NASA’s risk of exposure-induced death (REID) limit; exceeding it could effectively end an astronaut’s opportunity to fly again or, more gravely, irreversibly harm their long-term health. The accelerated trip makes it far easier for mission planners to keep each astronaut’s lifetime exposure below the agency’s career dose guidelines, allowing for more operational flexibility, greater opportunities for long-term spaceflight assignments, and lower long-term health risks. The reduced time in microgravity will also decrease bone mass loss^50^ before arrival at Mars. Astronauts lose bone mass at roughly 1% per month even with exercise, thus faster trajectories will maintain a few hundred extra grams of bone mass. Preserving bone mass is key to performing physically demanding tasks on the surface (setting up an ISRU plant) without heightened risk of fractures or debilitating muscle fatigue.

## Conclusion

Our results suggest that a 90-day Mars transit is technically feasible using chemical propulsion capabilities—specifically, the SpaceX Starship with anticipated orbital refueling and aerocapture capabilities. This markedly shorter transit time, relative to the standard six to nine month Hohmann windows, has the potential to reduce radiation exposure, minimize microgravity-related health risks, and lower the logistical burdens of prolonged spaceflight.

Nonetheless, several considerations highlight that this approach, while promising, is not free of challenges. First, the high launch cadence and large propellant demands require a level of operational maturity and ISRU that has yet to be demonstrated. Second, the limited publicly available data on Starship’s aerothermal performance introduces uncertainty into our atmospheric entry and deceleration simulations, which rely on simplified models and extrapolated heat flux estimates. Starship must also demonstrate the performance anticipated by this paper - the Raptor Vacuum ISP, structures mass and propellant mass must be in the range used by these calculations. Additionally, crew health factors-such as long-duration life support, microgravity countermeasures, and psychological well-being-must be thoroughly validated under the faster yet still significant interplanetary journey.

We therefore view this study as an initial framework that outlines the key elements required for a three-month Earth-Mars transfer, recognizing that more in-depth analyses (e.g., high-fidelity aerothermal simulations, updated propulsion performance data, and operational demonstrations) are necessary to fully validate the concept. With these caveats in mind, our findings indicate that the near-term capability for shorter transit time human Mars missions may be within reach, warranting further research.

## Data Availability

All data generated or analyzed during this study are included in this published article, or in the Github at https://github.com/jackSN8/mars-3-months.

## Appendix-A: Crew habitat mass estimates

The design of a crew habitat necessary to keep astronauts alive during this 90 day transit has not been revealed by SpaceX so it is necessary to estimate this. Skylab is the most relevant historical spacecraft as it involved adding a habitat to an existing rocket stage, similar to what will have to be done with Starship. Skylab took an S-IVB Saturn third stage which weighed 13t and added habitats that kept three astronauts alive without resupply for up to 84 days^51^ pushing the mass to 76t. Thus we can estimate a similar habitat for Starship might mass 63t.

We note that a team at the DLR German Aerospace Center^26^ did attempt to estimate the dry mass of a crew Starship, and estimated it at 204t. They calculate the structural mass of steel in Starship to be 40t, and then calculate the mass of the propellant tanks to be 20t, assuming they are spherical tanks inside the Starship structure, adding that to the structural mass. Starship does not have spherical tanks, and instead the steel outer structure is the outside of the tank^30^ (Fig. 9), so this paper ‘double counts’ the mass of the propellant tanks. The paper assumes cold gas helium is used for Starship’s RCS (Reaction Control System), but in reality it uses hot gas from the main tanks^52^. They add an extra 5.5t for this hypothetical helium RCS and the supporting tanks. The paper claims Starship uses PICA-X as its thermal protection system, which was in the design from 2016 to 2019, but was changed to ceramic tiles in 2019^53^. The paper uses the mass of Raptor 1 engines which are 2t. Raptor 1 last flew in 2021, and the under testing Raptor 3 engines weigh 1.5t^54^, reducing the mass for a 9-engine^13^ Starship by 4.5t, or 6-engine^53^ by 3t. These stated reasons are why we think that a crew Starship could mass significantly less than their estimate.

## Appendix B: Landing burn DV

The 500 m/s landing burn DV estimate is from Elon Musk’s 2017 IAC presentation^25^ where he claims Mars reentry bleeds 99% of the orbital energy off. This is 90% of the orbital velocity, thus we believe 500m/s is a reasonable estimate for landing burn DV. These values are for the 2017 BFS design, which is similar size and shape to the 2025 Starship design, thus will have similar terminal velocity and landing burn DV.

## References

[1] National Aeronautics and Space Administration (NASA). Human Exploration of Mars Design Reference Architecture 5.0 . Tech. Rep. NASA/SP-2009-566, NASA (2009) (accessed 31 Dec 2024).

[2] NASA. Mars exploration: NASA’s Mars page (2024). https://nssdc.gsfc.nasa.gov/planetary/planets/marspage.html (accessed 31 Dec 2024).

[3] Patel, Z. S., et al. Red risks for a journey to the red planet: The highest priority human health risks for a mission to Mars. *NPJ Micrograv.* **6**, 10.1038/s41526-020-00124-6 (2020). 10.1038/s41526-020-00124-6 PMC7645687 33298950

[4] Cucinotta, F. A., Kim, M.-H.Y., Chappell, L. J. & Huff, J. L. How safe is safe enough? Radiation risk for a human mission to mars. *PLoS One* **8**, e74988. 10.1371/journal.pone.0074988 (2013) (**Accessed: 2024-12-31.**). 24146746 10.1371/journal.pone.0074988 PMC3797711

[5] Lang, T. et al. Cortical and trabecular bone mineral loss from the spine and hip in long-duration spaceflight. *J. Bone Miner. Res.* **19**, 1006–1012. 10.1359/JBMR.040307 (2004) (**Accessed: 2025-02-24.**). 15125798 10.1359/JBMR.040307

[6] Chang-Díaz, D. F. Mars in 39 days? The VASIMR Plasma Engine. In *Presented at the DDP 31st Annual Meeting, July 13, 2013, Houston, TX* (2013). https://www.youtube.com/watch?v=-nyepvfuHho (accessed 31 Dec 2024).

[7] Borowski, S. K., McCurdy, D. R. & Packard, T. W. Nuclear Thermal Propulsion (NTP): A Proven, Growth Technology for “Fast Transit” Human Missions to Mars. Tech. Rep. NASA/TM-2014-218104, NASA Glenn Research Center (2014) (accessed 24 Feb 2025).

[8] Duplay, E., Bao, Z. F., Rosero, S. R., Sinha, A. & Higgins, A. Design of a rapid transit to Mars mission using laser-thermal propulsion. *Acta Astronaut.* **192**, 143–156. 10.1016/j.actaastro.2021.11.032 (2022) (**Accessed: 2025-02-24.**).

[9] National Aeronautics and Space Administration (NASA). NASA’s Journey to Mars: Pioneering Next Steps in Space Exploration. Tech. Rep. (2015) (accessed 31 Dec 2024).

[10] Stark, A. *et al.* ECI Modular Assembled Radiators for NEP Vehicles (MARVL), an Overview. In *Thermal & Fluids Analysis Workshop (TFAWS 2024)* (NASA Glenn Research Center, Cleveland, OH, 2024) (accessed 24 Feb 2025).

[11] Buenconsejo, R. S., Lal, B., Howieson, S. V., Behrens, J. R. & Kowal, K. Launch approval processes for the space nuclear power and propulsion enterprise. Tech. Rep. IDA Document D-10910, IDA Science & Technology Policy Institute (2019) (accessed 24 Feb 2025).

[12] SpaceX. Human Spaceflight: Mars Mission. https://www.spacex.com/humanspaceflight/mars/ (2024) (accessed 31 Dec 2024).

[13] SpaceX. SpaceX Starship Update 2024 (2024). https://x.com/SpaceX/status/1776669097490776563 (accessed 31 Dec 2024).

[14] Musk, E. Tweet from Elon Musk on January 17, 2020 (2020). https://x.com/elonmusk/status/1217989066181898240 (accessed 31 Dec 2024).

[15] SpaceX. SpaceX Mars Presentation 2016 (2016). https://web.archive.org/web/20160928040332/http://www.spacex.com/sites/spacex/files/mars_presentation.pdf (accessed 31 Dec 2024).

[16] Musk, E. & Dodd, T. Starbase tour and interview with Elon Musk (2022). https://everydayastronaut.com/starbase-tour-and-interview-with-elon-musk/ (accessed 31 Dec 2024).

[17] Musk, E. Tweet from Elon Musk (2024). https://x.com/elonmusk/status/1819781279828636041 (accessed 24 Feb 2025).

[18] Musk, E. Tweet from Elon Musk (2021). https://x.com/elonmusk/status/1409368907480113155 (accessed 23 Feb 2025).

[19] Musk, E. Tweet from Elon Musk (2023). https://x.com/elonmusk/status/1657249739925258240 (accessed 24 Feb 2025).

[20] Musk, E. Tweet from Elon Musk (2020). https://x.com/elonmusk/status/1309385646033842176 (accessed 23 Feb 2025).

[21] NASA Glenn Research Center. Chemical Equilibrium with Applications (CEA) (2025). https://cearun.grc.nasa.gov/ (accessed 23 Feb 2025).

[22] Rodríguez, J. L. C. & contributors. poliastro: Python library for interactive Astrodynamics (2024). https://github.com/poliastro/poliastro (accessed 31 Dec 2024).

[23] Kingdon, J. Mars-3-Months: Lambert solver and entry sims (2024). https://github.com/jackSN8/mars-3-months (accessed 31 Dec 2024).

[24] Wooster, P. D., Braun, R. D., Ahn, J. & Putnam, Z. R. Trajectory Options for Human Mars Missions. Tech. Rep., Massachusetts Institute of Technology and Georgia Institute of Technology (2009) (accessed 24 Feb 2025).

[25] SpaceX. Making Life Multiplanetary (2017). https://www.youtube.com/watch?v=tdUX3ypDVwIt=1388s (accessed 31 Dec 2024).

[26] Maiwald, V., Bauerfeind, M., Fälker, S., Westphal, B. & Bach, C. About the feasibility of SpaceX’s human exploration Mars mission scenario with Starship. *Sci. Rep.*10.1038/s41598-024-54012-0 (2024). 10.1038/s41598-024-54012-0 PMC11116405 38782962

[27] NASA. Apollo 11 Flight Journal-Day 9: Entry (1969). https://www.nasa.gov/history/afj/ap11fj/27day9-entry.html (accessed 31 Dec 2024).

[28] SpaceX. Human Spaceflight: Moon Mission (2024). https://www.spacex.com/humanspaceflight/moon/ (accessed 31 Dec 2024).

[29] SpaceX. Tweet from SpaceX on May 24th 2024 (2024). https://x.com/SpaceX/status/1793998848584794574?lang=en (accessed 07 Jan 2025).

[30] Federal Aviation Administration (FAA). Written Re-evaluation of the 2022 Final Programmatic Environmental Assessment for the SpaceX Starship/Super Heavy Launch Vehicle Program at the Boca Chica Launch Site in Cameron County, Texas (2022). https://www.faa.gov/media/27236 (accessed 31 Dec 2024).

[31] Musk, E. Tweet from Elon Musk on January 11, 2020 (2020). https://x.com/elonmusk/status/1215719463913345024 (accessed 31 Dec 2024).

[32] Veiga, A. 3 Seminário Internacional de Transporte e Desenvolvimento Hidroviário Interior: Development of an Inland Containerized Cargo Barge for LNG Transportation on Pará Basin. In *3 Seminário Internacional de Transporte e Desenvolvimento Hidroviário Interior* (Aprumar Marine Systems, 2023) (accessed 31 Dec 2024).

[33] Oxygen-Thermophysical Properties: Chemical, Physical, and Thermal Properties of Oxygen-O2. https://www.engineeringtoolbox.com/oxygen-d_1422.html (accessed 31 Dec 2024).

[34] Ring Watchers. Stacking Diagrams (2024). https://ringwatchers.com/diagrams/stacking-diagrams/ (accessed 31 Dec 2024).

[35] Hackaday. How Different Are SpaceX Thermal Tiles From the Space Shuttle’s? (2024) https://hackaday.com/2024/02/14/how-different-are-spacex-thermal-tiles-from-the-space-shuttles/ (accessed 24 Feb 2025).

[36] Emissivity Table: Pyrometry and Thermography (2024). https://www.transmetra.ch/images/transmetra_pdf/publikationen_literatur/pyrometrie-thermografie/emissivity_table.pdf (accessed 31 Dec 2024).

[37] NASA. Thermal Protection Materials Branch-Coatings (2023). https://www.nasa.gov/general/thermal-protection-materials-branch-coatings/ (accessed 24 Feb 2025).

[38] Jr, J. M. Thermal Conductivity and Electrical Resitivity of type 316 stainless steel. Tech. Rep. NASA-TM-X-53428, National Aeronautics and Space Administration (1965) (accessed 31 Dec 2024).

[39] Berger, E. Inside Elon Musk’s plan to build one Starship a week-and settle Mars (2020). https://arstechnica.com/science/2020/03/inside-elon-musks-plan-to-build-one-starship-a-week-and-settle-mars/ (accessed 31 Dec 2024).

[40] National Oceanic and Atmospheric Administration (NOAA), National Aeronautics and Space Administration (NASA) & United States Air Force. U.S. Standard Atmosphere, 1976. Tech. Rep., NOAA, NASA, and U.S. Air Force (1976) (accessed 24 Feb 2025).

[41] Jet Propulsion Laboratory (JPL). Martian Atmosphere and Its Effects on Propagation. Tech. Rep., NASA Jet Propulsion Laboratory (Year Unknown) (accessed 24 Feb 2025).

[42] Zhiwen, L., Lei, Z., Liang, L., Bin, H. & Haitao, Y. Performance and characteristics analysis on the Starship aerodynamic configuration. *Acta Aerodyn. Sin.* **40** (2022). (accessed 31 Dec 2024).

[43] Sutton, K. & Jr., R. A. G. A General Stagnation-Point Convective-Heating Equation for Arbitrary Gas Mixtures. Tech. Rep. NASA TR R-376, NASA Langley Research Center (1971) (accessed 24 Feb 2025).

[44] European Space Agency (ESA). Study Report: [High Lift over Drag Earth Reentry]. Tech. Rep., European Space Agency (2009) (accessed 23 Feb 2025).

[45] Brykina, I. G. & Egorova, L. A. Approximation formulas for the radiative heat flux at high velocities. *Fluid Dyn.* **54**, 562–574. 10.1134/S0015462819040037 (2019).

[46] Zubrin, R. M., Baker, D. A. & Gwynne, O. Mars direct: A simple, robust, and cost effective architecture for the space exploration initiative. In *AIAA Aerospace Sciences Meeting and Exhibit, AIAA-91-0328* (American Institute of Aeronautics and Astronautics (AIAA), 1991).

[47] Zeitlin, C. et al. Measurements of energetic particle radiation in transit to mars on the mars science laboratory. *Science* **340**, 1080–1084. 10.1126/science.1235989 (2013). 23723233 10.1126/science.1235989

[48] Hu, S., Monadjemi, S., Barzilla, J. & Semones, E. Acute radiation risks tool (ARRT) development for the upcoming human exploration missions. *Space Weather*. **18**, e2020SW002586. 10.1029/2020SW002586 (2020).

[49] Townsend, L. W. & Fry, R. J. M. Radiation protection guidance for activities in low-Earth orbit. *Adv. Space Res.* **30**, 957–963. 10.1016/s0273-1177(02)00160-6 (2002) (**Accessed: 2024-12-31.**). 12539765 10.1016/s0273-1177(02)00160-6

[50] LeBlanc, A. et al. Bone mineral and lean tissue loss after long duration space flight. *J. Musculoskelet. Neuronal Interact.* **1**, 157–160 (2000) (**Accessed: 2025-02-24.**). 15758512

[51] NASA. Skylab: A guidebook (1973). https://ntrs.nasa.gov/api/citations/19730025070/downloads/19730025070.pdf (accessed 15th May 2025).

[52] Musk, E. & Dodd, T. Starship RCS engineering change (2022). https://www.youtube.com/watch?v=t705r8ICkRw, https://www.youtube.com/watch?v=3Ux6B3bvO0w (accessed 31 Dec 2024).

[53] SpaceX. Starship Update 2019 (2019). https://www.youtube.com/watch?v=sOpMrVnjYeYt=10s (accessed 31 Dec 2024).

[54] SpaceX. Tweet from SpaceX on Aug 3, 2024 (2024). https://x.com/SpaceX/status/1819772716339339664 (accessed 31 Dec 2024).

